# Wristbands in Home-Based Rehabilitation—Validation of Heart Rate Measurement

**DOI:** 10.3390/s22010060

**Published:** 2021-12-23

**Authors:** Magdalena Jachymek, Michał T. Jachymek, Radosław M. Kiedrowicz, Jarosław Kaźmierczak, Edyta Płońska-Gościniak, Małgorzata Peregud-Pogorzelska

**Affiliations:** 1 Cardiology Clinic, Pomeranian Medical University, ul Powstańców Wielkopolskich 72, 70-111 Szczecin, Poland; radkied@wp.pl (R.M.K.); jar.kazmierczak@o2.pl (J.K.); edytaplonska@life.pl (E.P.-G.); m1peregud@gmail.com (M.P.-P.); 2 Physiotherapy Department, University of Social Sciences, ul. Sienkiewicza 9, 90-113 Lodz, Poland; michaljachymek@gmail.com

**Keywords:** smart band, heart rate, wearable, validation

## Abstract

The possibility of using a smartwatch as a rehabilitation tool to monitor patients’ heart rates during exercise has gained the attention of many researchers. This study aimed to evaluate the accuracy and precision of the HR measurement performed by two wrist monitors: the Fitbit Charge 4 and the Xiaomi Mi Band 5. Thirty-one healthy volunteers were asked to perform a stress test on a treadmill. Their heart rates were recorded simultaneously by the wristbands and an electrocardiogram (ECG) at 1 min intervals. The mean absolute error percentage (MAPE), Lin’s concordance correlation coefficient (LCCC), and Bland–Altman analysis were calculated to compare the precision and accuracy of heart rate measurements. The estimated validation criteria were MAPE < 10% and LCCC < 0.8. The overall MAPE and LCCC of the Fitbit were 10.19% (±11.79%) and 0.753 (95% CI: 0.717–0.785), respectively. The MAPE and LCCC of the Xiaomi were 6.89% (±9.75) and 0.903 (0.886–0.917), respectively. The precision and accuracy of both devices decreased with the increased exercise intensity. The accuracy of wearable wrist-worn heart rate monitors varies and depends on the intensity of training. Therefore, the decision to use such a device as a heart rate monitor during in-home rehabilitation should be made with caution.

## 1. Introduction

Recent advances in mobile sensor technology have increased the popularity of wrist-worn fitness trackers (wristbands, smart bands, and smartwatches). The main purpose of these devices is to monitor training progress and daily physical activity. Commercially available wristbands offer continuous measurement of the heart rate (HR), step count, energy expenditure, saturation, and sleep duration. More advanced medical algorithms include detecting atrial fibrillation, estimating heart rate variability to assess the autonomic nervous system, and continuous glucose monitoring [1]. The newest models may have a one-lead ECG option.

The popularity of such devices gives a unique opportunity to acquire a large amount of data about the patients, which can be analyzed in medical research. The expense of professional medical biosensors was usually an obstacle in the way of running medical research on a large scale. Commercial wearables give the patients an insight into their results, involve them in the process and make them more self-conscious. The data are collected in the natural setting of the patient, which allows the patient to assess their lifestyle habits. Therefore, making it possible to find connections between behavior, physical activity, sleep quality, and diseases.

The possibility of using commercially available wrist-worn monitors in medical research was a topic of many validation studies and clinical trials. An analysis published in 2018 included 423 devices, and the top five brands were Fitbit, Xiaomi, Apple, Garmin, and Samsung. The authors identified 81 studies in the MEDLINE database, including 61 validation/reliability studies and 20 data collection studies. Fifty-five ongoing or planned studies were registered in the Clinical Trials database, including 6 validation and 45 data collection studies [2]. It shows that many researchers saw smartwatches as a valuable tool with which to conduct their research, even though it is not professional medical equipment.

Wrist monitors have recently gained considerable interest as potential rehabilitation tools because they may increase the availability of rehabilitation and decrease medical staff involvement. The patients can take responsibility for the entire rehabilitation program after instruction on training, monitoring their vital signs, and obtaining regular feedback from a nurse, physiotherapist, or doctor. A randomized controlled trial evaluating smart bands used in a home-based rehabilitation demonstrated that home-based rehabilitation is non-inferior to traditional outpatient cardiac rehabilitation [3]. Mobile sensor technology is still new and continuously developing. Every year the producers launch new models of wrist-worn devices. Commercially available smartwatches are not professional medical equipment. The producers do not have to provide validation studies on the accuracy and precision of particular wristbands. They also often disclose that it is not supposed to be used as medical or research equipment in the manual (for example, Fitbit). However, the accuracy of HR monitoring is crucial during exercise and for estimating energy expenditure. HR sensors in smart bands are based on photoplethysmography, an optical technique that measures blood volume changes in the microvascular bed of the tissue with each heartbeat [4].

This study aimed to evaluate the accuracy and precision of HR measurement performed by two popular wrist-worn monitors, the Fitbit Charge 4 (Fitbit) and Xiaomi Mi Band 5 (Xiaomi), and assess whether patients can use these monitors to monitor HR during home-based rehabilitation.

## 2. Materials and Methods

### 2.1. Materials

Fitbit wristbands are widely used in medical research [2]. An advantage of Fitbit monitors is the opportunity to store training data not only on the mobile Fitbit app but also in the cloud in the web-based app, which allows a third party, such as a physiotherapist or trainer, to access the fitness data. This may improve compliance and facilitate remote monitoring.

The Xiaomi MI Band 5 is a very popular tool because of its competitive price. Xiaomi connects with Google FIT, a mobile app that collects data from many devices and applications. The functionality of both devices is compared in Table 1.

### 2.2. Study Protocol

Study participants performed a treadmill stress test according to the Bruce protocol. The Bruce protocol consists of several 3 min stages. In each stage, the speed and incline increase. During exercise, all participants wore the Xiaomi wristband on the right hand and the Fitbit wristband on the left hand. Exercise was preceded by a 3 min resting period (stage 0) and followed by a 3 min recovery period.

Stress tests were performed on a treadmill. The ECG was analyzed by, HearTwave^®^ IIsoftware, Cambridge Heart Inc., Wilmington, MA, USA. ECG data were continuously monitored through 12 electrodes placed on the chest with Manson–Likar modification. The heart rate was automatically calculated from the ECG with a ≤5% readout error. The ECG HR was a gold standard to which wristbands’ HR measurements were compared.

Stress tests were stopped when the submaximal HR limit was achieved. The HR limit was automatically calculated by the HearTwave software using the patient’s age. With the patient’s consent, the stress test was continued until the maximum HR limit (220—age) was reached or until maximum exhaustion. During stress tests, at rest, and during recovery, HR was simultaneously manually recorded from the ECG and both wristbands every minute.

The study protocol was approved by the local medical university ethics board (nr: KB-0012/150/2020).

### 2.3. Participants

Thirty-one healthy Caucasian volunteers were enrolled in the study. Inclusion criteria were age >18 years, no prior cardiovascular disease, and the willingness and ability to perform a stress test. The study cohort was an age-diverse group (18–70 years) of 21 men and 10 women. All were physically active on a regular basis. Basic information on the participants and stress test results are presented in Table 2.

Some measurements were lost because of technical problems. In 29 cases, the stress test was terminated after the HR limit was achieved. Exercise was stopped in one case because of high blood pressure and in another because of technical problems with the treadmill. Three participants wore only the Fitbit wristband.

### 2.4. Statistical Analyses

The statistical analyses were performed using Statistica 13.3 and IBM SPSS Statistics software.

To test the accuracy of devices for each pair of HR measurements, absolute error (AE) and absolute percentage error (APE) were calculated according to the following formulas:

AE = |HREKG − HR device|

APE = |(AE/HREKG) × 100%|

The mean absolute error (MAE) and mean absolute percentage error (MAPE) were calculated. An MAPE of <10% was considered reliable [5,6].

A multi-regression model was created to test the relationship between sex, age, weight, and height. Differences in AE between groups according to sex and stage of exercise were compared using the Mann–Whitney U test, the Kruskal–Wallis test, and post hoc tests. A *p* value of <0.05 was considered statistically significant.

To test the reliability of both fitness trackers, Lin’s concordance correlation coefficient (LCCC) was estimated. An LCCC of >0.8 was considered good agreement [7].

The Bland–Altman analysis was performed to analyze the agreement between each device and the ECG recordings.

All normally distributed data are presented as mean ± standard deviation (SD). Nonparametric data are presented as medians and the interval between the minimum and maximum values.

## 3. Results

A total of 556 pairs of data were obtained for Fitbit, and 509 pairs were obtained for Xiaomi. The overall MAPE of the Fitbit device was 10.19% (±11.79%), which did not meet the validation criteria. The MAPE of Xiaomi was lower (6.89% ± 9.75%). Because the AE for both devices did not follow a normal distribution, the median AE was compared between devices. The overall; overall male; and stage 1, 2, and 4 median AEs were significantly lower for Xiaomi compared with Fitbit (Table 3).

When each device was analyzed separately (data not shown), no differences were observed in AE according to sex (*p* = 0.125 for Fitbit, *p* = 0.098 for Xiaomi). Fitbit’s AE was significantly higher in stage 4 compared with that in stage 0 and recovery. Xiaomi’s AE did not differ between stages. Because of the small number of measurements in stage 6 (only two participants achieved this stage), they were not included in separate calculations.

A multiple regression model revealed no relationship between the MAPEs of Fitbit and Xiaomi and the covariates (sex, age, height, and weight).

**Figure 1:**
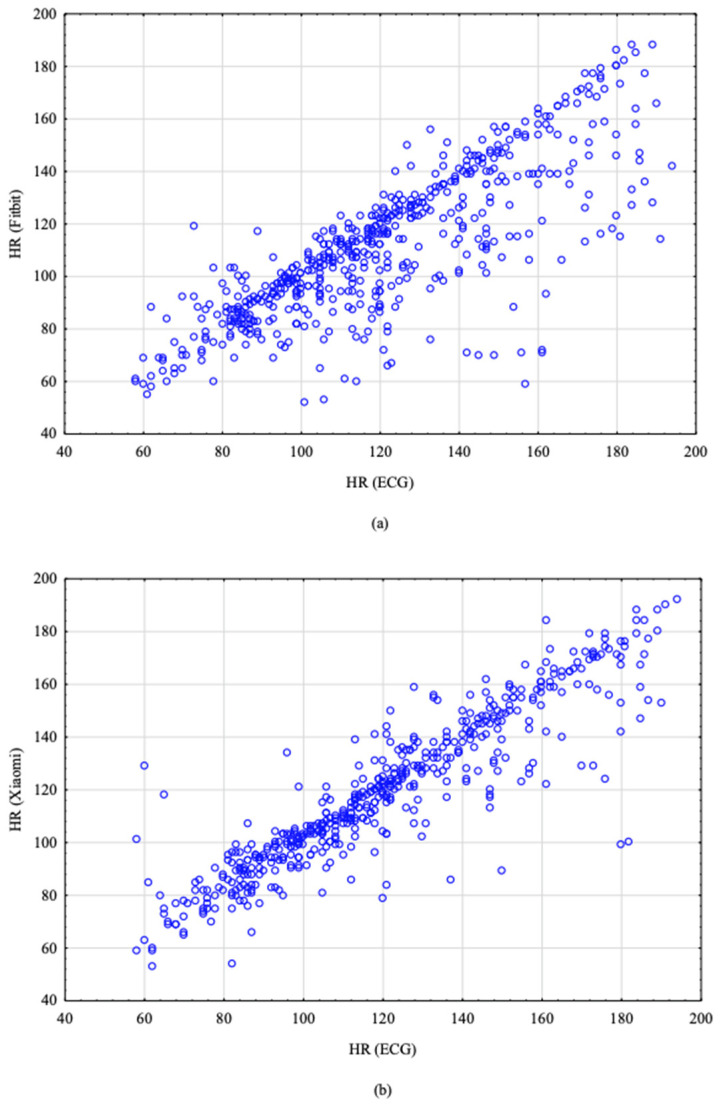
Scatterplots of pairs of HR measurements. (**a**) HR readings obtained from the ECG and Fitbit; (**b**) HR readings obtained from the ECG and Xiaomi.

Figure 1 displays the scatterplots of pairs of HR measurements (ECG and Fitbit, ECG and Xiaomi). In Fitbit’s scatterplot, most observations create a line, which means they correlate well, but there are a lot of observations beneath the main line. This means that many of Fitbit’s readouts were lower than the actual HR. In Xiaomi’s scatterplot, the observations are symmetrically distributed.

The LCCCs of the Fitbit and Xiaomi HR measurements were 0.753 (95% CI: 0.717–0.785) and 0.903 (0.886–0.917), respectively. Again, the Fitbit readouts correlated less well with the ECG and were beneath the validation criteria in all stages. The LCCC of both devices decreased with increased intensity of physical activity (Table 4). The LCCC in stage 5 should be interpreted with caution, as there were too few observations in this stage.

**Figure 2:**
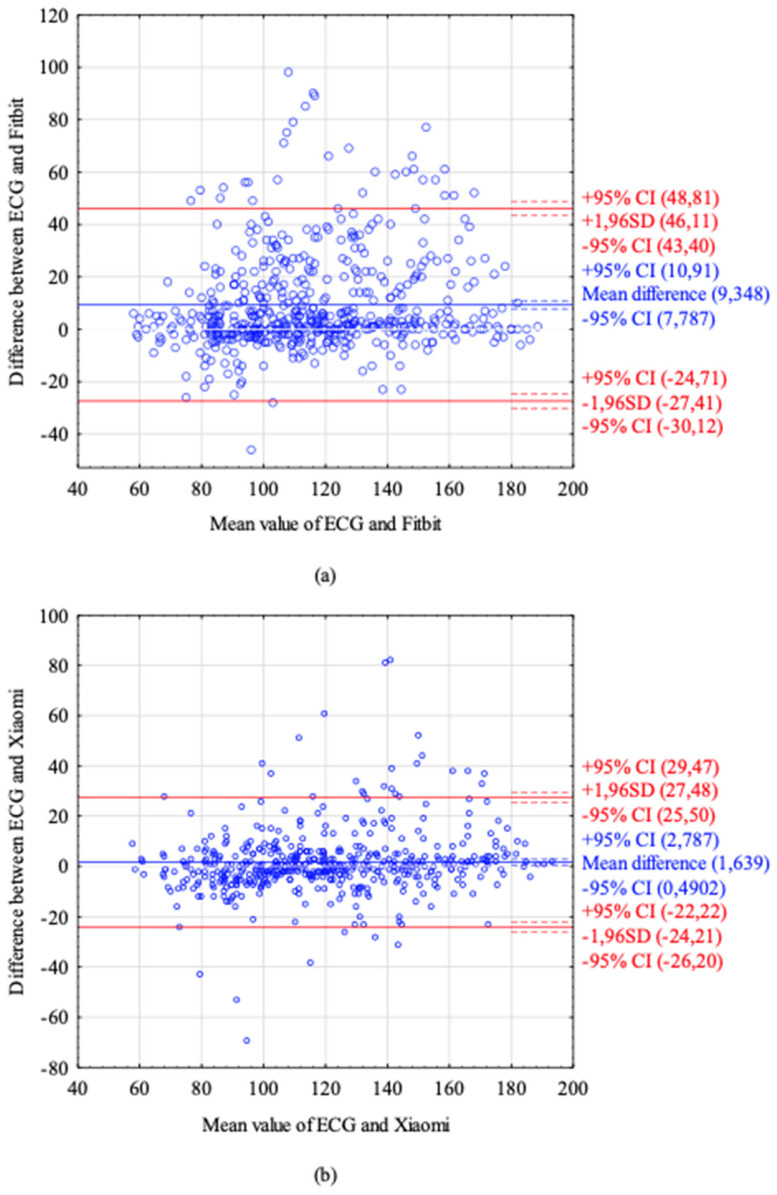
Bland–Altman plots. (**a**) Bland–Altman plot of HR readings from the ECG and Fitbit. (**b**) Bland–Altman plot of HR readings from the ECG and Xiaomi. The blue line represents mean difference between the ECG HR and device HR. The red lines represent ± 1.96 SD (standard deviation), the dotted lines represent ± 95% CI (confidence interval).

The Bland–Altman analysis revealed that Fitbit tended to underestimate HR values, with a mean difference of 9.348 BPM. This confirms the tendency already spotted on scatterplots. Xiaomi did not show any tendency in HR estimation, with a mean difference of 1.639 BPM. The Bland–Altman plots are presented in Figure 2. Dots outside the red lines correspond to extreme error values.

## 4. Discussion

This study validated the HR measurements of two wrist-worn monitors: the Fitbit Charge 4 and the Xiaomi Mi Band 5. This is the first study to evaluate these two models.

In our study, the Fitbit device did not fulfill the presumed validation criteria. The overall MAPE and the MAPE for each stage of exercise were >10% and were lower only at rest and during recovery. However, median values better represent nonparametric data than mean values, and both smart bands had a median APE of <10%. We used mean values because they are typically used in validation studies. Fitbit’s overall LCCC and the LCCC at each stage were <0.8. The Xiaomi Mi Band 5 had superior performance, with an overall MAPE of 6.89% and an LCCC of 0.903.

The available studies evaluating Fitbit devices have mostly assessed older models, such as the Fitbit Charge 2, Fitbit Charge HR, Fitbit Blaze, and Fitbit Surge. Methodologies have differed between studies: reference methods typically involve an ECG [5,6] or the Polar chest strap [7,8,9], which is a validated tool with which to estimate HR.

Most studies evaluating Fitbit devices have reported a lower MAPE than our study, ranging from 2.38% [7] to 6.2% [9]. The accuracy and reliability of fitness trackers decrease with increased exercise intensity [7,9,10], reaching an MAPE 3 of 8.24% and an LCCC of 0.12 during intense exercise on an ergometer [10]. Conversely, one study observed the lowest agreement during moderate exercise, which improved with more intense physical activity [5].

Correlations between the gold standard and device readings differ depending on the activity type. For example, when comparing cycling, walking, running, arm raises, planks, and lunges, the Pearson’s product-moment correlation was the lowest while performing lunges (r = 0.28) and planks (r = 0.26, Fitbit Charge HR) [6] or while on an elliptical trainer (LCCC = 0.58) and the highest on a treadmill (LCCC = 0.76, Fitbit Blaze) [8].

Most validation studies are conducted with healthy volunteers. People with cardiovascular disease may have lower heart rate accuracy because of several factors, such as peripheral atherosclerosis and increased arterial stiffness associated with hypertension. The Fitbit Blaze was evaluated in this population during various activities (at rest, cycling, and walking on a treadmill), and the results were as follows: MAPE of 6.6% and LCCC of 0.88 at rest; MAPE of 8.6% and LCCC of 0.76 on a treadmill; and MAPE of 8.4% and LCCC of 0.72 during cycling. These results appear to be comparable with those obtained in a healthy population [11].

Fewer data are available for Xiaomi fitness trackers. In our study, the Xiaomi data were generally correlated more strongly with the ECG data, and the Xiaomi device showed a lower error rate than the Fitbit device; however, Xiaomi also exhibited inferior outcomes during intense exercise. In a study by Hsueh-Wen et al. [12], the overall MAPE of the Xiaomi Mi Band 2 was 8.85%. The authors divided the participants into two groups, young and elderly (>65 years), and they did not identify a significant difference in the reliability between groups. However, the LCCC was 0.73 in both age groups, and this was below the designated threshold. The LCCC also differed depending on the activity type and reached the lowest values in the younger group during cycling (0.29) and exercise on an elliptical trainer (0.32).

We did not identify any co-factors that altered the reliability, such as sex, age, height, weight, and BMI, which is consistent with previous results [8,9,12]. Shcherbina et al. reported a higher error rate for males than for females across all evaluated devices, including the Fitbit Surge [13]. We observed higher error rates and lower correlation coefficients among females, but the differences between sexes for both devices were not statistically significant. In both devices, some extreme errors were identified, and errors occurred more often with higher HR values. Chow et al. [12] observed that extreme readings were unpredictable, unexpected, and transient. In our experience, the most important precaution taken to avoid extreme readings was properly adjusting the wristband to the size of the wrist.

In home-based rehabilitation, the recommended activity is either walking or running because of the lack of equipment required. In our study, the intensity of the effort increased quite quickly; this led to a rapid increase in heart rate, and the maximum HR was reached within several minutes. During normal training, the HR is lower than the HR achieved in the exercise test; in addition, the HR should be maintained within the recommended range throughout the training period. After stabilizing the HR, the accuracy of the device could improve, but extreme values may still occur. Continuing a workout beyond the recommended HR threshold may be dangerous to the patient.

The models with the ECG option may offer an improvement in HR accuracy in the future. Several models with ECG monitoring with atrial fibrillation detection mode are available on the market (for example, Apple Watch Series 4–7, Samsung Galaxy Watch Active 2, Samsung Galaxy Watch 2–3, Fitbit Sense). Although producers claim that these are not medical or scientific devices in terms of use, they have been cleared by Food and Drug Administration (FDA) for heart rate monitoring and atrial fibrillation detection, but only as an informative tool, not as a diagnostic one [14].

Home-based rehabilitation programs monitored with smart bands were evaluated in several populations. In the population of patients with pulmonary arterial hypertension, an increase in the number of steps performed improved the distance in the 6 min walk test and the quality of life [15]. In a population of patients with metabolic syndrome, a 12-week marching training program combined with weekly support from a qualified nurse lowered blood pressure, but it had no effect on waist circumference, fasting glycemia, lipid levels, or body composition. In this study, a high dropout rate was observed; of the 53 initially included participants, only 20 finished the program. Those who resigned from the study were older, and resignation most often resulted from difficulties using a smartphone and the mobile application [16].

Operating smart devices may be the main obstacle facing rehabilitation programs for older patients. The average age of patients undergoing rehabilitation is increasing. An intervention consisting of an online program in combination with an activity monitor (accelerometer) and coaching for 12 weeks improved the quality of life of older patients (average age over 60 years) [17], but it is difficult to transfer these conclusions to the general population. Participants were volunteers who were likely more motivated to change and had higher education levels and intellectual abilities than the population on average. According to a large telephone survey conducted on 1349 residents of Australia, smartwatch users were less likely to have low education and low physical activity levels or to be unemployed [18].

Our study has several limitations. Firstly, the participants differed in age, but most of them were 20–30 years old, so it is not possible to determine the reliability in older patients on the basis of our results. However, a previous study observed no difference in reliability between younger and older patients [12]. Secondly, according to calculations made by other authors, 8 [12] to 25 [8,19] pairs of data are the minimum number necessary to gain statistical significance when calculating the LCCC. Because the duration of the stress test was different for each participant, far fewer observations were obtained at higher exercise intensities. Some authors suggested that a different methodology, such as acquiring data every second, could be superior to observations collected every minute because more data could be collected in the same period [6]. Thirdly, the exercise protocol used in our study was designed to reach the maximum HR limit in a short period of time; it is possible that after stabilizing the HR, both devices would be more reliable.

## 5. Conclusions

Both devices revealed a certain degree of error, which was more considerable in the Fitbit device. At rest, the Fitbit HR error was just above the validation threshold, but during exercise, it increased rapidly. The correlation between the ECG and Fitbit readouts fell with increasing exercise intensity. The Xiaomi device performed better but considerably decreased in precision during exercise. Considering our results and the results of previous studies, the researchers and doctors should decide to use wrist-worn monitors in home-based rehabilitation with caution. The manufacturers do not recommend these wrist monitors for medical or scientific measurement. The reliability of both devices decreased during exercise, and at high HR values, some extreme errors occurred. Underestimating HR values may be dangerous to patients. Not all devices provide the opportunity to control training progress remotely. We believe that home-based rehabilitation programs using wristbands as HR monitors may be suitable for patients with low cardiovascular risk who are highly motivated and have high intellectual abilities. The wristband should be used only as a self-control tool which is not a substitute for a traditional monitored rehabilitation. Proper instructions on using such devices and interpreting the results are crucial for safety.

## Figures and Tables

**Table 1:** Features and specifications of the Fitbit Charge 4 and Xiaomi MI Band 5.

Functions	Fitbit Charge 4	Xiaomi Mi Band 5
Price	EUR 149.95	~EUR 40
Battery life	Up to 7 days	Up to 14 days
GPS	Yes	No
Photoplethysmography (PPG) heart rate (HR) monitor	Yes (measured in 1 s intervals)	Yes
Syncing	Bluetooth, near-field communication (NFC)	Bluetooth
Operating system	Android, iOS	Android, iOS
Sleep Tracking	Yes	Yes
All-day activity tracking	Steps, distance, calories, activity time	Steps, calories, activity time
Training mode	Yes	Yes
Automatic exercise recognition	Yes	No
Waterproof	Up to 50 m	Up to 5 atm
Application	Yes	Yes
Payments	Yes	No
Smartphone notifications	Yes	Yes
Google FIT App	No	Yes
Internet application	Yes	No

**Table 2:** Participants’ characteristics and stress test results.

	Total	Female	Male
n	31	10	21
Age (years)	28 (18–71)	27.5 (23–54)	32 (18–71)
Height (cm)	177.7 (±14.62)	168 (±4.5)	181.9 (±6.3)
BMI (kg/m^2^)	23.5 (20.1–37.1)	22 (20–23)	24 (21–37.1)
Peak HR (BMP)	164.7 (±19.9)	161.1 (±13.8)	166.5 (±22.4)
%HR MAX	88.8 (±8.5)	85.1 (±6.8)	90.6 (±8.7)
METs	14.3 (±3)	12.7 (±1.2)	15 (±3.2)

n: number of participants; BMI: body mass index; Peak HR: maximum HR achieved during the stress test; BPM: beats per minute; %HR MAX: percentage of calculated maximal HR achieved during the stress test; METs: estimated metabolic equivalents achieved during the stress test.

**Table 3:** Comparison between Fitbit and Xiaomi error rates.

Subgroup (N Measurements)		Fitbit		Xiaomi		*p*
		Absolute Error (BPM)	Absolute Percentage Error (%)	Absolute Error (BPM)	Absolute Percentage Error (%)	
Total n = 557	Mean	12.84 ± 16.55	10.19 ± 11.79	7.99 ± 10.61	7.99 ± 10.61	
	Median	6 (0–98)	5.17 (0–63)	4 (0–82)	3.95 (0–115)	<0.0001
Male n = 391	Mean	12.59 ± 16.70	9.92 ± 11.67	7.74 ± 10.90	6.73 ± 10.35	
	Median	5 (0–90)	4.92 (0–63.01)	4 (0–82)	3.85 (0–115)	<0.001
Female n = 166	Mean	13.46 ± 16.21	10.83 ± 12.09	8.82 ± 9.58	7.40 ± 7.46	
	Median	7 (0–98)	6.08 (0–62.42)	5 (0–44)	4.84 (0–38.58)	0.0528
Stage 0 n_F_ = 93, n_x_ = 84	Mean	7.56 ± 8.45	8.69 ± 9.06	8.08 ± 10.65	10.58 ± 17.14	
	Median	5 (0–54)	5.33 (0–47.37)	5 (0–69)	5.67 (0–115)	0.86
Stage 1 n_F_ = 90, n_x_ = 81	Mean	12.81 ± 12.04	12.20 ± 11.27	4.58 ± 4.73	4.80 ± 5.32	
	Median	8 (0–46)	8.51 (0–63.01)	3 (0–28)	3.53 (0–34.15)	0.001
Stage 2 n_F_ = 93, n_x_ = 84	Mean	11.78 ± 13.96	10.17 ± 11.66	6.68 ± 7.33	5.99 ± 5.77	
	Median	6 (0–57)	5.38 (0–50)	4.5 (0–34)	4.37 (0–23.13)	0.0012
Stage 3 n_F_ = 90, n_x_ = 81	Mean	14.71 ± 19.60	10.61 ± 13.36	7.38 ± 8.01	5.53 ± 5.91	
	Median	5 (0–98)	3.88 (0–62.42)	4 (0–29)	2.88 (0–23.01)	0.14
Stage 4 n_F_ = 70, n_x_ = 67	Mean	21.47 ± 21.67	13.16 ± 12.31	8.73 ±11.38	5.52 ± 7.28	
	Median	16 (1–79)	10.11 (0.57–53.02)	4 (0–51)	2.53 (0–37.22)	0.0012
Stage 5 n = 25	Mean	21.6 ± 26.23	11.97 ± 12.88	12.72 ±16.19	7.41 ± 9.92	
	Median	6 (0–85)	3.33 (0–54.49)	5 (0–61)	2.76 (0–40.67)	0.4
Stage 6 n = 6	Mean	1.67 ± 4.08	0.89 ± 2.18	46.5 ± 30.32	25.7 ± 16.87	
	Median	0 (0–10)	0 (0–5.35)	42.5 (13–82)	23.6 (7.22–45.05)	0.017
Recovery n_F_ = 90, n_x_ = 81	Mean	9.2 ± 14.84	7.15 ± 10.51	8.33 ± 8.81	7.03 ± 7.81	
	Median	4 (0–90)	3.36 (0–55.9)	6 (0–41)	4.42 (0–39.58)	0.38

n_F_: number of measurements from Fitbit; n_x_: number of measurements from Xiaomi; BPM: beats per minute. Mann–Whitney U test was used to compare absolute percentage error medians; *p* < 0.05 was considered statistically significant.

**Table 4:** Lin’s concordance correlation coefficient (LCCC).

Stage of Exercise	Fitbit (LCCC, 95% CI)	Xiaomi (LCCC, 95% CI)
Total	0.753 (0.717–0.785)	0.903 (0.886–0.917)
Stage 0	0.757 (0.655–0.831)	0.675 (0.542–0.774)
Stage 1	0.3 (0.139–0.446)	0.912 (0.867–0.942)
Stage 2	0.408 (0.250–0.545)	0.795 (0.704–0.861)
Stage 3	0.228 (0.075–0.371)	0.730 (0.612–0.816)
Stage 4	0.176 (0.04–0.305)	0.660 (0.518–0.766)
Stage 5	0.051 (0.161–0.259)	0.455 (0.209–0.647)
Recovery	0.651 (0.517–0.753)	0.851 (0.783–0.899)
Female	0.688 (0.605–0.756)	0.887 (0.842–0.919)
Male	0.774 (0.743–0.809)	0.906 (0.887–0.922)

LCCC: Lin’s concordance correlation coefficient; CI: confidence interval.

## Data Availability

Data presented in this study are openly available in Zenodo repository at DOI: 10.5281/zenodo.5075267.
